# Age-Related Nuclear Translocation of P2X6 Subunit Modifies Splicing Activity Interacting with Splicing Factor 3A1

**DOI:** 10.1371/journal.pone.0123121

**Published:** 2015-04-13

**Authors:** Juan Ignacio Díaz-Hernández, Álvaro Sebastián-Serrano, Rosa Gómez-Villafuertes, Miguel Díaz-Hernández, María Teresa Miras-Portugal

**Affiliations:** 1 Department of Biochemistry and Molecular Biology, Veterinary School, Complutense University of Madrid, Madrid, Spain; 2 Instituto de Investigación Sanitaria del Hospital Clínico San Carlos, IdISSC, Madrid, Spain; CNRS UMR7275, FRANCE

## Abstract

P2X receptors are ligand-gated ion channels sensitive to extracellular nucleotides formed by the assembling of three equal or different P2X subunits. In this work we report, for the first time, the accumulation of the P2X6 subunit inside the nucleus of hippocampal neurons in an age-dependent way. This location is favored by its anchorage to endoplasmic reticulum through its N-terminal domain. The extracellular domain of P2X6 subunit is the key to reach the nucleus, where it presents a speckled distribution pattern and is retained by interaction with the nuclear envelope protein spectrin α2. The *in vivo* results showed that, once inside the nucleus, P2X6 subunit interacts with the splicing factor 3A1, which ultimately results in a reduction of the mRNA splicing activity. Our data provide new insights into post-transcriptional regulation of mRNA splicing, describing a novel mechanism that could explain why this process is sensitive to changes that occur with age.

## Introduction

P2X purinergic receptors (P2XRs) are ligand-gated ion channels activated by extracellular ATP. At the plasma membrane, functional P2XRs are formed by the assembling of three equal or different P2X subunits named P2X1 to P2X7. All P2X subunits share a common structure composed of two transmembrane domains joined by a large extracellular loop with both N- and C-terminal ends positioned to the cytoplasmic side of the membrane [1]. At the CNS, the signaling mediated by P2XRs has been linked with many physiological processes including pain, inflammation, neuronal differentiation and neurogenesis among others [2].

In the hippocampus it has been described that P2XRs modulate neurotransmitter release [3], facilitate the induction of long-term potentiation (LTP) [4], regulate axonal growth and branching [5] and also interact with other membrane receptors [1]. In this location P2X6 assembles with other P2X subunits, mainly P2X2 and P2X4, forming functional heterotrimeric receptors at the plasma membrane of granular cells [6]. However, a specific function associated with P2X6 subunit currently remains unknown. Despite the expression of P2X6 subunit being relatively abundant in the brain, either at mRNA level [7] or protein level [6], it fails to form stable homomeric receptors [8]. This inability has been associated with its capacity to remain anchored to the endoplasmic reticulum (ER) membrane by the hydrophobic N-terminal end. In addition, this retention avoids its trafficking [9] unless another P2X subunit interacts and guides the P2X6 subunit to the plasma membrane [10,11].

Pre-mRNA splicing is an essential cellular process which removes introns and joins the coding sequences of a nascent transcript giving rise a mature mRNA molecule [12]. Splicing of pre-mRNA occurs in two sequential trans-esterification reactions and takes place in a large ribonucleoprotein complex known as the spliceosome, composed of the dynamic assembly of five small nuclear ribonucleproteins (snRNP) and additional splicing factors [12]. The snRNP responsible for recognizing the intron branch point (BS) is the snRNP U2, which is composed of several protein complexes, one of them is the splicing factor 3A (SF3A), a heterotrimeric protein complex [13]. SF3A1 is one of the components of SF3A complex that, although it is released from U2 snRNP before the second transesterification, it is essential for the initial recognition of the BS [14]. The spliceosome components are localized inside the nucleus, both in a dynamic structure called nuclear speckles or, diffusely outside this structure in the nucleoplasm [15]. It is worth noting that although the recruitment of the splicing components inside speckles has been associated with a decrease of the splicing activity [16], neither the function nor the mechanism of formation of nuclear speckles has been well established yet [17]. In this sense, it has also been demonstrated that changes in the splicing of transcripts could be crucial in relevant physiopathological processes such as aging or neurodegeneration [18].

Here we show that the retention of P2X6 subunit in the ER allows it to reach the nucleus. Our aim is to understand how this subunit reaches the nuclear structure and its physiological role. The extracellular region of P2X6 subunit exhibits a protein interacting domain that could be a good candidate to target it in the nucleus. Once at the nuclear envelope, P2X6 subunit interacts with spectrin α2. In addition, P2X6 binds to the splicing factor 3 subunit A1 (SF3A1) inside the nucleus, resulting in a reduction of the splicing activity. Importantly, we report for the first time that a P2X subunit has a different function from its ability to assemble ligand-gated ion channels at the plasma membrane, and that the spliceosome activity can be modulated by an ionotropic receptor subunit.

## Materials and Methods

### Ethics statement

All animal procedures were carried out at the Universidad Complutense de Madrid in accordance with European and Spanish regulations (86/609/CEE; RD1201/2005) following the guidelines of the International Council for the Laboratory Animal Science. C57BL/6 mice were obtained from in-house breeding. The protocol was approved by the Committee of Animal Experiments of the Complutense University of Madrid. All surgery was performed under isofluorane anesthesia, and all efforts were made to minimize suffering.

### Cell culture

Primary cultures of hippocampal neurons were prepared as previously described [19]. Briefly, the pregnant dam was sacrified by cervical dislocation as described by the AVMA Guidelines on Euthanasia, and E18 mouse embryos were isolated from the uterus. The hippocampus was dissected and dissociated from mouse embryos using the Papain Dissociation System (PDS; Worthington Biochemical Corp.). Neurons were seeded at a density of 10,000 cells/cm^2^ on poly-L-lysine-coated coverslips (1 mg/ml) (Sigma-Aldrich). After plating, neurons were maintained in Neurobasal A medium supplemented with 1% B-27, 0.5 mM glutamine, 1 mM pyruvate, 100 U/ml penicillin, and 100 mg/ml streptomycin (all from Life Technologies). Cytosine β-D-arabinofuranoside (Sigma-Aldrich) was added at a final concentration of 5μM after two days in culture. When neurons were kept in culture for more than 9 days, coverslips were transferred to plates containing astrocyte monolayers in neuronal culture medium (Neurobasal A supplemented with B-27, Life Technologies) added 24 hours before culture.

Neuro-2a neuroblastoma cell line (N2a) was also used in the present study. Cells were obtained from ATCC (ATCC CCL-131). Cells were cultured in DMEM (high glucose) (Sigma-Aldrich) supplemented with Glutamax, penicillin/streptomycin (Life technologies), and 10% heat-inactivated FBS (Euroclone) and grown at 37°C in a humidified atmosphere containing 5% CO_2_.

### Generation of P2X6 expression plasmids and cell transfection

The P2X6-YFP construct was generated by PCR from IMAGE cDNA clone for human P2X6 (cDNA clone MGC: 34460 IMAGE: 5176046, Source BioScience) with KOD polymerase (EMD Millipore) and cloned between KpnI and ApaI (Promega Biotech) sites of the multiple cloning site of the pYFP-N1 vector (Clontech). The P2X6-myc construct was generated by PCR from P2X6-YFP and sub-cloned between KpnI and ApaI sites of the multiple cloning site of the pcDNA3.1-myc vector (Clontech). Plamids containing different P2X6 subregions were generated by PCR with KOD polymerase and cloned in pcDNA3.1-myc for tag detection. pCAG-GFP plasmid, which expresses green fluorescent protein under the control of chicken β-actin promoter and rabbit β-globin poly (A) signal (abbreviated as EGFP) [20], was used as electroporation control vector.

Transient transfections of N2a cells were carried out using Lipofectamine 2000 (Life Technologies) following the manufacturer’s instructions. Transfection of hippocampal neurons was performed 24 hours after plating using Lipofectamine 2000 (9 μl) and 3 μg of DNA. The transfection mix was removed after 2 h and the neurons were washed and maintained for 3 DIV until processed.

### Design and cloning of small hairpin RNAs (shRNAs) targeted against P2X6 subunit

Plasmid construction and shRNA plasmid design was performed as previously described [5]. The shRNA oligonucleotides were designed according to a previously reported rational design protocol and shRNA target sequence 5’-TGCTCAAGCTCTATGGAAT-3’ for the P2X6 mRNA was selected. The sequence specificity was confirmed by a BLAST analysis of the human, mouse and rat P2X6 receptors. Synthetic forward and reverse 64-nucleotide oligonucleotides (Sigma Genosys) were designed annealed and inserted into the BglII/HindIII sites of the pSUPER.neo.GFP vector (OligoEngine) following the manufacturer’s instructions. The concomitant expression of green fluorescent protein (GFP) from this vector allowed transfected cells to be identified by fluorescence. The plasmids generated express 19-bp plus 9-nucleotide stem-loop shRNAs targeted against P2X6 mRNA. As control the firefly luciferase-targeted oligonucleotide 5’-CTGACGCGGAATACTTCGA-3’ (shRNA Luc) was used.

### Generation of luciferase reporter plasmids to quantify splicing activity

The TPI-Luciferase/Renilla construct I (TPI-I) was generated by cloning human triose phosphate isomerase exons 6 and 7 (85 and 38 nt, respectively) plus intron 6 (139 nt) [21] in frame with the firefly luciferase gene (cloned by PCR from pGL4.10, Promega) in the MCS1 of pBI-CMV1 bidirectional vector (Clontech). *Renilla* luciferase gene (cloned by PCR from pGL-4.74, Promega) was also subcloned in MCS2 of pBI-CMV1 as internal control. The TPI-Luciferase/Renilla construct II (TPI-II) is identical to construct I except that site-directed mutagenesis was used to remove an in-frame stop codon in the intron and add a G at position 6 in TPI exon 7. Constructions I and II were mutated in the ATG codon of firefly luciferase in order to avoid alternative internal initiation of translation. Constructs I’ and II’ were obtained by site-directed mutagenesis of I or II respectively to inactivate the 5’ splice site.

### Luciferase Reporter Assay

N2a cells were plated on 24-well plates coated with poly-L-lysine the day before transfection (cells reached 80% confluence the day of transfection). Plasmid constructions I, II, I’ or II’ were transfected and 24 hour later cells were reseed at 20.000cel/cm^2^ to induce differentiation by serum deprivation. Cells were harvested after 4 days in Passive Lysis Buffer (Promega) and assayed for luciferase activity. Firefly luciferase and *Renilla* luciferase activities were measured sequentially using the Dual-Luciferase Reporter Assay System (Promega). Firefly luciferase activity was normalized according to *Renilla* and expressed as relative luminescence units (RLU). These vectors function as follows: when the splicing activity is blocked in neural cells, for example after 6 hours treatment with the spliceosome inhibitor isoginkgetin 33 μM, the ratio firefly/*renilla* luciferase measured in cells transfected with vector I decreases, whereas in cells transfected with vector II this ratio increases. As internal control to normalize data between independent experiments, we used vectors I’ and II’, where the ratio firefly/*renilla* luciferase do not change in any case.

### *In utero* electroporation

Morning of the day of the appearance of the vaginal plug was defined as embryonic day (E) 0.5. At E14.5 pregnant C57BL/6 female mice were anesthetized by continuous inhalation of Isoflurane (Baxter). The abdomen was opened and the uterine horns exposed. The DNA solution (1 μg/μL each plasmid; P2X6-YFP, CAG GFP) containing 0.03% fast green in PBS was injected into one lateral ventricle of each embryo using a pulled glass micropipette. The head of each embryo was placed between tweezer type electrodes (CUY 650 P5 Nepa GENE) and five square electric pulses (38V, 50 ms) were passed at 1 sec intervals using an Electro Square Porator ECM 830 (BTX, Harvard Apparatus). The wall and skin of the abdominal cavity were sutured and the embryos were allowed to develop normally until birth (P0).

### Tissue processing for immunohistochemistry and immunofluorescence

All P0 animals were anesthetized using a mix of ketamine (80–200 mg/kg) and xylazine (7–20 mg/kg) diluted in PBS and administered as a single intraperitoneal injection. Mice were perfused transcardially with 0.1 M phosphate-buffered saline (PBS; pH 7.4) followed by cold 4% paraformaldehyde in PBS (PFA; pH 7.4) (Sigma-Aldrich). The perfused brains were removed and post-fixed in 4% PFA at 4°C overnight. After washing in PBS for 30 min, fixed brains were cryoprotected in 30% sucrose in PBS. The samples were embedded in OCT compound (Sakura) and frozen using dry ice. Finally, 25 μm free-floating coronal sections were cut with a cryostat (CM1950, Leica Microsystems).

Adult mice were sacrificed by cervical dislocation, decapitated and the forebrain removed. Brains were fixed in 4% paraformaldehyde and cryoprotected in 30% sucrose solution. Sagittal or coronal sections (30 μm) were cut on a cryostat, collected free-floating and stored in a solution of 30% ethylene glycol, 30% glycerol and 0.1 M PBS at -20°C until processed.

### Immunohistochemistry techniques

Tissue sections were stained free-floating using the biotin–avidin-peroxidase method, with 3,3′-Diaminobenzidine tetrahydrochloride (DAB) as a chromogen. Endogenous peroxidase was inactivated by incubating sections in a solution of 0.3% hydrogen peroxide in PBS for 30 min. Brain sections were pretreated for 1 h with 1% bovine serum albumin (BSA) (Sigma-Aldrich), 5% FBS, and 0.2% Triton X-100 (Sigma-Aldrich) in PBS, and subsequently incubated with rabbit polyclonal antibodies against P2X6 (Alomone Labs, 1/50), P2X4 (Alomone Labs, 1/500), and/or P2X2 (Alomone Labs, 1/200) receptors. Finally, brain sections were incubated with avidin-biotin complex using Elite Vectastain kit (Vector Laboratories). Chromogen reactions were performed with 3,3′-Diaminobenzidine tetrahydrochloride (Sigma*FAST* DAB, Sigma Aldrich) and 0.003% H_2_O_2_ for 10 min. Sections was mounted on glass slides and coverslipped using FluorSave (Calbiochem).

Staining for SA-β-Gal was performed as previously described [22]. Shortly, frozen sections were washed in PBS twice and immersed in X-Gal staining solution (60mM citric acid/Na_2_HPO_4_ buffer, 5mM K_4_[Fe(CN)_6_]3H_2_O, 5mM K_3_[Fe(CN)_6_], 150mM NaCl, 2mM MgCl_2_ and 1 mg/ml X-gal, pH = 6). X-gal (5-bromo-4-chloro-3-indolyl-/3-D-galactopyranoside) was made up as a stock solution of 20 mg/ml in *N*,*N*-Dimethylformamide (Sigma Aldrich). After incubation in the dark at 37°C for 36–48 h, sections were washed in PBS, and immunostained with DAB as described before.

Transmitted light images were acquired in an inverted microscope (Eclipse TE200, Nikon) with DFC310FX camera (Leica Microsystems GmbH) using Leica Aplication Suite (v4.1). Sections were photographed with Plan 4× dry objective lens NA = 0.1 and insets with Plan S-Fluor 40× dry objective lens NA = 0.90 (Nikon) at room temperature.

### Immunofluorescence analysis

For hippocampal immunofluorescence studies, tissue slices were washed in PBS, blocked for 1 h at room temperature with 5% FBS and 1% BSA in PBS containing 0.2% Triton-X 100 (blocking solution) and then incubated at 37°C for 1 h or overnight at 4°C with primary antibodies diluted in blocking solution. Fluorescent-tagged secondary antibodies (in PBS containing 2% FBS) were incubated at 37°C for 1 h, and sections were counterstained with 4',6-diamidino-2-phenylindole dihydrochloride hydrate (DAPI) (Molecular Probes) and mounted in FluorSave.

The following primary antibodies were used at the dilutions indicated: rabbit anti-GFP (Life Technologies, 1:500), rabbit anti-SF3A1 (Aviva Biosystems, 1:100) and mouse anti-GFP (Roche, 1:400). Goat anti-rabbit and anti-mouse secondary antibodies, conjugated with Alexa 594 and 488, respectively (Molecular Probes), were used at 1:500. Before immunostaining with anti-SF3A1 antibody, sections were treated for antigen unmasking with 2 N HCl for 30 min.

For immunofluorescence detection in cell culture, the cells were seeded on poly-L-lysine treated coverslips and fixed with 4% paraformaldehyde. Nonspecific binding was blocked with 1% BSA, 5% FBS and 0.2% Triton X-100 in PBS. Cells were then incubated with primary antibodies at the following dilutions: rabbit anti-P2X6 (Alomone Labs, 1:200), mouse anti-c-myc (Life Technologies, 1/1000), mouse anti-α-tubulin (Sigma Aldrich, 1/1000), mouse anti-Map-2 (Sigma Aldrich, 1/500), mouse anti-NeuN (Chemicon, 1/500), mouse anti SC35 (Sigma Aldrich, 1/1000), mouse anti fibrillarin (Santa Cruz, 1/200),rabbit anti-GFP (Life Technologies, 1/500) and mouse anti β-III-tubulin (Promega, 1/1000), for 1 h at room temperature. Then coverslips were washed three times with 1% BSA in PBS and incubated with Alexa-Fluor-488, Alexa-Fluor-594 or Alexa Fluor-694-conjugated secondary antibodies (Life technologies, 1:400) and nuclei were stained with DAPI. Coverslips were mounted ProLong Gold antifade solution (Life Technologies). Confocal images were acquired with a TCS SPE microscope from Leica Microsystems equipped with a Plan Fluor 10× dry objective lens NA = 0.30, 40 X Apochromat NA = 1.15 oil objective lens and 63× Apochromat NA = 1.3 oil objective lens (Leica Microsystems GmbH) at room temperature. For the detection of DAPI, 405 nm laser line was used. For Alexa Fluor 594, the 561 nm laser line was used. For Alexa Fluor 488, the 488 nm laser line was used. Pictures were acquired using the Leica software LAS AF v2.2.1 software (Leica Microsystems GmbH) and representative slices converted to TIFF files using ImageJ 1.47d (NIH).

### Electron microscopy

For electron microscopy, sections were post-fixed in 2% OsO_4_ for 1 h, dehydrated, embedded in araldite, and mounted in Formvard-coated slides using plastic cover-slips. After polymerization, selected areas were photographed, trimmed, re-embedded in araldite, and re-sectioned at 1–2 μm. These semithin sections were re-photographed and re-sectioned in ultrathin sections. The ultrathin sections were examined in a Jeol 301 electron microscope (JEOL Ltd.).

### Immunoprecipitation

Nuclear fraction of tissue samples (400 μl) was obtained with ProteoExtract Subcellular Proteome Extraction Kit (Calbiochem) according to manufacturer instructions Samples were incubated in presence of P2X6 (Chemicon), spectrin α2 (Santa Cruz Biotechnology) antibodies (2 μg each) or mouse IgG as control for immunoprecipitation, all previously immobilized on 50 μl MagSi-ProteinG beads (Magna Medics) at room temperature for 1h. Antigen-antibody complexes were separated with magnets, washed three times with PBS and resuspended in 1x Laemmli’s buffer. Samples were heated at 95°C for 10 min and separated on 10% SDS-PAGE. For 2D PAGE, the immunoprecipitation samples were resuspended in suitable buffer (7 M Urea, 2 M thiourea, 2% CHAPS, 1% ampholytes pH 3–11, and 10 mM DTT).

### Western blot analysis

Protein extracts from freshly-dissected mouse hippocampi were obtained by tissue homogenization in ice-cold lysis buffer: 20 mM HEPES (pH 7.4), 100 mM NaCl, 10 mM NaF, 1% Triton X-100, 1 mM Na_3_VO_4_, 10 mM EDTA, and Complete Protease Inhibitor Cocktail (Roche). Protein extracts from cells were obtained by lysis in NP-40 extraction buffer (Tris HCl 50mM, NaCl 150mM, Nonidet NP-40 1%, 10 mM NaF, 1 mM Na_3_VO_4_, Complete Protease Inhibitor Cocktail). Total protein extracts (20 μg) were electrophoresed on 10% Tris-Glycine SDS-PAGE gel and transferred to nitrocellulose membranes (Whatman). Antibodies used were: P2X6 (Chemicon, 1:200), P2X2 (1:200), P2X4 (1:1000), GFP (1:1000), c-myc (1:1000), spectrin α2 (1:200), histone 2b (Cell Signalling, 1:1000) and α-tubulin (Sigma Aldrich, 1:10000). Secondary antibodies used were monoclonal goat anti-mouse (Dako, 1:5000), and polyclonal goat anti-rabbit (Dako, 1:1000), followed by enhanced chemoluminescence detection (Perkin Elmer, USA). Images were captured with ImageQuant LAS 500 (GE Healthcare Life Sciences) and analysed using ImageJ software (v1.47d).

### 2D PAGE

Immunoprecipitated samples were resuspended in sample buffer (7 M Urea, 2 M thiourea, 2% CHAPS, 1% ampholytes pH 3–11, and 10 mM DTT) and incubated for 1h under agitation to remove magnetic particles. Samples were loaded onto 7 cm pH 3–11 nonlinear DryStrips (GE Healthcare), which had been rehydrated applying 50V for 20°C during 10h with sample buffer. The first dimension with denaturing isoelectric focusing was run using the following program: 500 V for 15 min, 1000 V for 30 min, 1000–5000 V gradient for 1.5 h and final step of 5000V for 2h. Strips were soaked in reducing buffer (6 M urea, 100 mM Tris-HCl at pH 8.0, 30% glycerol (v/v), 2% SDS (w/v), and 2% DTT (w/v)) for 12 min followed by another 5 min in the same buffer, which was supplemented with 2.5% iodoacetamide. The second SDS-PAGE (10% T, 2.6% C) dimension was run for 1 h at 100V per gel at 25°C with adecuate protein weight marker (Precision Plus Dual Colours, Bio-Rad). Protein spots were visualized by staining with Coomassie Brilliant Blue. This procedure was realized by Proteomics Unit at Universidad Complutense de Madrid.

### Mass Spectrometry Analysis of Protein Spots

Once the 2D gels were scanned, the spots showing differences in protein expression and considered to be of interest were aligned with the Colloidal Coomassie Blue profile, manually excised using pipet tips and transferred to microcentrifuge tubes. Samples selected were in-gel reduced, alkylated and digested with trypsin [23]. Briefly, spots were washed twice with double distilled water, shrunk 15 min with 100% acetonitrile and dried under vacuum in a Savant SpeedVac for 30 min. The samples were then reduced with 10 mM dithioerytritol in 25 mM ammonium bicarbonate for 30 min at 56°C, and subsequently alkylated with 55 mM iodoacetamide in 25 mM ammonium bicarbonate for 15 min in the dark. Finally, samples were digested with 12.5 ng/mL sequencing grade bovine trypsin (Roche Molecular Biochemicals) in 25 mM ammonium bicarbonate (pH 8.5) overnight at 37°C. After digestion, the supernatant was collected and 1μL was spotted onto a matrix-assisted laser desorption/ionization (MALDI) target plate and allowed to air-dry at room temperature. Next, 0.4 μL of 3 mg/mL of R-cyano-4-hydroxy-transcinnamic acid matrix (Sigma) in 50% acetonitrile were added to the dried peptide digest spots and allowed again to air-dry at room temperature. MALDI-TOF MS analyses were performed in a 4800 Proteomics Analyzer MALDI-TOF/TOF mass spectrometer (Applied Biosystems). The instrument was operated in reflector mode, with an accelerating voltage of 20 000 V. All mass spectra were calibrated externally using a standard peptide mixture (Sigma). Peptides from the auto digestion of the trypsin were used for the internal calibration. MALDI-TOF MS analysis produces peptide mass fingerprints and the peptides observed can be collected and represented as a list of mono isotopic molecular weights with an S/N greater than 20. The suitable precursors for MS/MS sequencing analyses were selected and fragmentation was carried out using the CID on (atmospheric gas was used) 1 KV ion reflector mode and precursor mass window 610 Da. The plate model and default calibration were optimized for the MS/MS spectra processing.

Peptide sequence searching was performed in batch mode and the peptides (with +1 charge) were identified by filtering the peaks using the software GPS Explorer v3.6 (Applied Biosystems) with MASCOT 1.9 using mouse database.

MALDI-TOF and peptide searching were performed by Proteomics Unit at Universidad Complutense de Madrid.

### Bioinformatics and statistical analysis

P2X receptors protein sequences were obtained from NCBI (P2X1, P51575; P2X2, Q9UBL9; P2X3, P56373; P2X4, Q99571; P2X5, Q93086; P2X6, O15547 and P2X7, Q99572), aligned using ClustalX algorithm in CLC Genomics Workbench v3.6.5 (CLC bio) and compared with full PFAM database plugin v22.0.

NLS sequences were searched using NLSMapper website (http://nls-mapper.iab.keio.ac.jp/cgi-bin/NLS_Mapper_form.cgi) with a cut-off score of 5.0 and entire region to search bipartite NLS [24].

Unless otherwise stated data are shown as mean values ± standard error of the mean (s.e.m). The numbers of mice per group used in each experiment are annotated in the corresponding Figure legends as n. All experiments shown were reproduced 2–5 independent times. Figures and statistical analyses were generated using GraphPad Prism 6 (GraphPad Software) Results were analyzed by un-paired Student´s *t*-tests or ANOVA with Dunett post-test. The statistical test used and P values are indicated in each Figure legend. P ≤ 0.05 was considered statistically significant. *P ≤ 0.05, **P ≤ 0.01 and NS, not significant.

## Results

### P2X6 subunit is present in the nucleus of neurons

**Fig 1:**
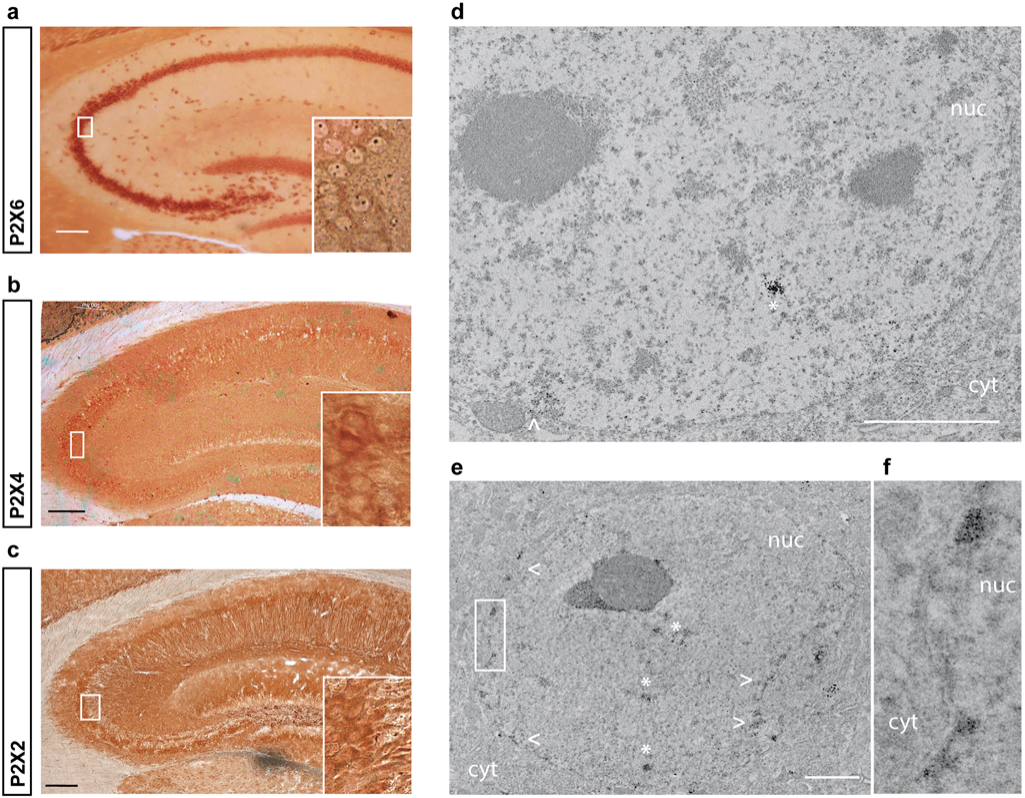
Expression of P2X6 subunit in adult mice hippocampus. (**a-c**) Immunohistochemical analysis of hippocampus sections from 8 month-old mice. Cells were labeled with antibodies against P2X6 (**a**), P2X4 (**b**) and P2X2 (**c**) subunits. Insets depict enlarged views (40X magnification) of CA3 hippocampal region showing a dotted pattern for P2X6 into the nucleus in addition to cytoplasmic staining (**a**), somatodendritic stainining for P2X4 (**b**) and axonal and cytoplasmic staining for P2X2(**c**). Scale bar 200 μm. (**d-f**) Immuno-electron microscopy analysis of a CA3 hippocampal neuron from 8 months-old wild type mouse labeled with anti-P2X6 antibody. The staining is observed both in the cytoplasm (cyt, white arrows) and into the nucleus (nuc, white asterisks). Scale bar 2 μm. (**d-e**) Inset of the area indicated in (**e**) showing a 3.5 X magnification of this indicated area. P2X6 positive regions are mainly located in patches added to the inner nuclear membrane (**e**).

In mice, P2X6, P2X4 and P2X2 subunits are mainly expressed in the cell body of the hippocampal neurons, especially in CA1, CA2, CA3 areas and granular layer from dentate gyrus (DG) (Fig 1a–1c). A more detailed analysis of these regions showed that while P2X2 and P2X4 subunits presented a somatodendritic distribution (Fig 1b and 1c and insets), P2X6 subunits also exhibited a nuclear location especially in cells from CA3 area (Fig 1a inset). The nuclear location of P2X6 was confirmed by ultrastructural analysis by electron microscopy (Fig 1d–1f). As shown in Fig 1d, P2X6 subunits were mainly found inside the nucleus in an aggregated form both in nucleoplasm (Fig 1e) or associated to the inner membrane of the nuclear envelope (Fig 1f). It is worth noting that nuclear accumulations of P2X6 subunit resemble interchromatin granule clusters with a variable size and irregular shape, similar to the nuclear structures known as speckles [17].

**Fig 2:**
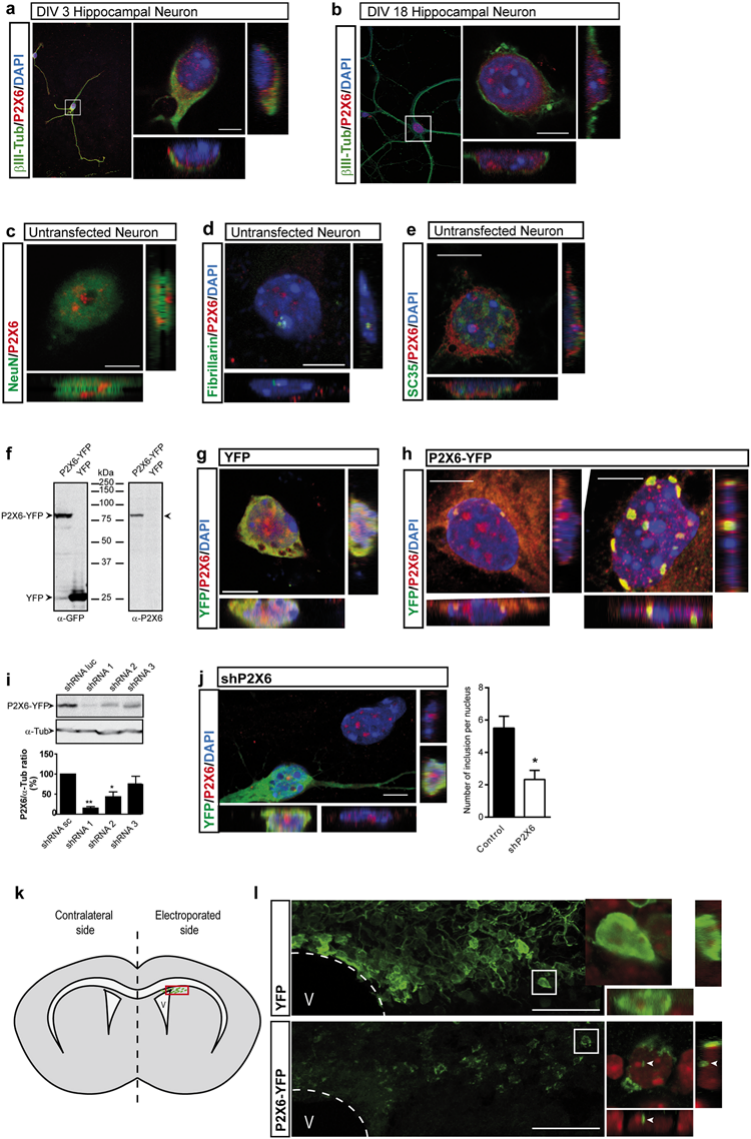
Subcellular distribution of native P2X6 subunits in hippocampal neurons *in vitro*. (**a,b**) Immunofluorescence images of 3(**a**) and 18(**b**) days-old primary cultures of hippocampal neurons labeled with antibodies against P2X6 and β-III Tubulin. Nuclei were counterstained with DAPI. Confocal image and orthogonal views of hippocampal neurons show that P2X6 immunostaining is localized in ER compatible location and inside the nucleus, and shows a more dotted pattern as days in culture passed. Scale bar 5 μm. (**c-e**) Immunofluorescence images 16–18 days-old primary cultures of hippocampal neurons labeled with antibodies against P2X6 and NeuN (**c**), fibrillarin (**d**) or SC35 (**e**). Nuclei were counterstained with DAPI. Confocal image and orthogonal views of hippocampal neurons show that P2X6 immunostaining is localized in ER compatible location and inside the nucleus without co-localization with NeuN (**c**), fibrillarin (**d**) or SC35 (**e**). Scale bar 5 μm. (**f**) Western blot of protein extracts from neuroblastoma cells transfected with either P2X6-YFP chimeric protein or YFP vector and labelled with antibodies against GFP and P2X6. (**g, h**) Immunofluorescence of hippocampal neurons transfected with either empty vector YFP (**g**) or P2X6-YFP (**h**). Confocal image and orthogonal views in (**h**) shows that P2X6 and YFP staining are distributed in cytosol, but are predominantly accumulated close to the nuclear membrane and also inside the nucleus without co-localization with DAPI. Scale bar 5 μm. (**i**) P2X6 expression was knocked down in neuroblastoma cells transfected with P2X6-YFP using three specific shRNAs. shRNA against luciferase (shRNA Luc) was used as negative control. Protein extracts were immunoblotted with anti-GFP antibody in order to characterize the efficiency of each shRNA. (mean±s.e.m, n = 3 independent experiments, * P<0.05, ** P<0.01 unpaired Student's t-test). (**J**) Endogenous P2X6 expression was knocked down in hippocampal neurons transfected with shRNA 1, showing less nuclear inclusions than non-transfected neurons. Scale Bar 10 μm. Bar graph represents the number of nuclear inclusions detected with anti-P2X6 antibody in neuronal nucleus (mean±s.e.m., n = 30 cells *P<0.01 unpaired Student’s t-test). (**k, l**) Cartoon of intraventricular electroporation of E14.5 embryo mice (**k**). Confocal microphotography of P0 mice brain from both YFP or P2X6-YFP electroporated mice showing the nuclear and perinuclear location of P2X6 in P2X6-YFP transfected mice (**l**). V, ventricle. Scale Bar 50 μm.

The nuclear accumulation of P2X6 subunit was also observed *in vitro* in primary cultures of hippocampal neurons. Immunodetection of native P2X6 subunits in neurons showed a prominent somatic location, although a clear staining inside the nucleus was also observed (Fig 2a and 2b). Noticeably, the formation of intranuclear P2X6 clusters increased as days in culture passed (Fig 2a and 2b), and it was not related either to chromatin nor to nucleolar structures (Fig 2c and 2d). Interestingly, despite the nuclear localization of P2X6 subunit presented a speckled distribution pattern, this subunit did not colocalize with the specific marker of nuclear speckles sc-35 (Fig 2e).

To study the nuclear translocation of P2X6 subunit, we generated a plasmid encoding human P2RX6 (NM_005446.3) fused with the yellow fluorescence protein (P2X6-YFP) (Fig 2e). When cultured hippocampal neurons were transfected with these constructs, accumulation of fluorescence spots inside or surrounding the nucleus were observed in neurons transfected with P2X6-YFP (Fig 2g), but not in those transfected with an empty vector (YFP) (Fig 2f). Nuclear localization of native P2X6 was significantly reduced when neurons were transfected with shRNA against P2X6 (Fig 2h and 2i).

Finally, to discard that the results obtained in mice were due to a nonspecific interaction of the antibodies used, we resorted to the *in utero* electroporation technique with the plasmid previously generated (Fig 2j). The results showed that cortical neurons expressing P2X6-YFP presented an accumulation of fluorescence spots inside the nucleus that were not observed in neurons electroporated with YFP (Fig 2k).

### Nuclear location of P2X6 subunit is increased with aging but is not correlated with senescence

**Fig 3:**
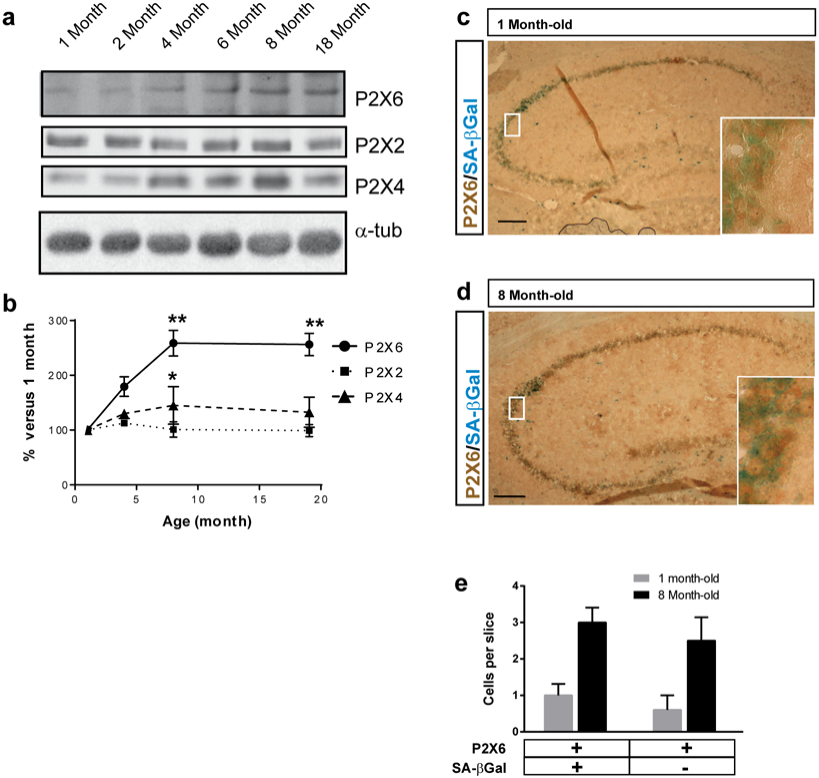
Age-dependent expression of P2X6 is not related with senescence. (**a**) Western blot analysis for expression of P2X2, P2X4 and P2X6 proteins in mice (from 1 to 18 month-old) (**b**) Graph represent P2X6, P2X2 and P2X4 levels relative to 1 month-old mice. Levels for P2X6 expression is on average 2.58±0.14-fold higher, P2X4 subunit only increases 1.45±0.19-fold at 8 month, and P2X2 level remains invariable (mean±s.e.m, n = 7 mice, * P<0.05, ** P<0.01 One-way ANOVA with Dunnett post-hoc test using 1 month-old mice data as control). (**c,d**) Immunohistochemical analysis of brain section from 1 (**c**) and 8 months-old (**d**) mice. Immunohistochemistry against P2X6 subunits (brown) and detection of senescence-associated β-galactosidase activity (SA-βGal, blue) in hippocampal slices. Scale bar 200 μm. Insets depict enlarged views (40X magnification) of delimited area. (**e**) Quantification of both P2X6 and/or SA-βGal positive cells in hippocampal slices from 1 month-old and 8 month-old mice (mean±s.e.m., n = 3 mice, P = 0.72 unpaired Student’s t-test between same age).

Taking into account that nuclear accumulation of P2X6 subunits increased according to the number of days in culture, we wonder whether this phenomenon was related to neuronal maturation or neuronal senescence. To address this study, firstly, we analyzed the expression levels of P2X6 subunit in mice hippocampus at different ages (1, 2, 4, 8 and 18 months-old). Interestingly we observed that the hippocampal expression of P2X6 subunit increases with age, reaching its maximal level at the age of 8 months-, maintaining this expression level up to the older ages analyzed (18 months old) (Fig 3a). On the other hand, when the expression levels of P2X2 and P2X4 subunits, no significant changes were observed, except a slight but significant increase of P2X4 subunit at 8 months-old mice that disappears in 18 months-old animals (Fig 3a). Once established the P2X6 subunit expression levels as a function of time, two reference time points were selected to analyze whether or not the nuclear localization of P2X6 is affected by aging, 1 month-old as levels were minimum, and 8 months-old, when levels were at its maximum. Interestingly, the number of neurons that presented a nuclear P2X6 staining was significantly higher in 8 months-old mice, being up to three times higher than in 1 month-old mice (S1a Fig). As expected, no significant changes were observed between 8 and 18 months-old mice regarding the number of neurons that presented nuclear P2X6 staining (S1b Fig).

Secondly, considering the relationship between the nuclear translocation of P2X6 and the age of the animal, we wanted to know whether this process could be involved in neuronal senescence. To address this point we counted the number of senescent neurons, identified as β-galactosidase positive cells [25], which also presented nuclear P2X6 expression. The results obtained showed that in both 1 and 8 months-old mice the quantity of senescent neurons with nuclear location of P2X6 subunit was similar to the number of non-senescent neurons that possessed nuclear inclusions of P2X6 subunits (Fig 3c–3e).

### Extracellular domain of P2X6 subunit is responsible for nuclear location

**Fig 4:**
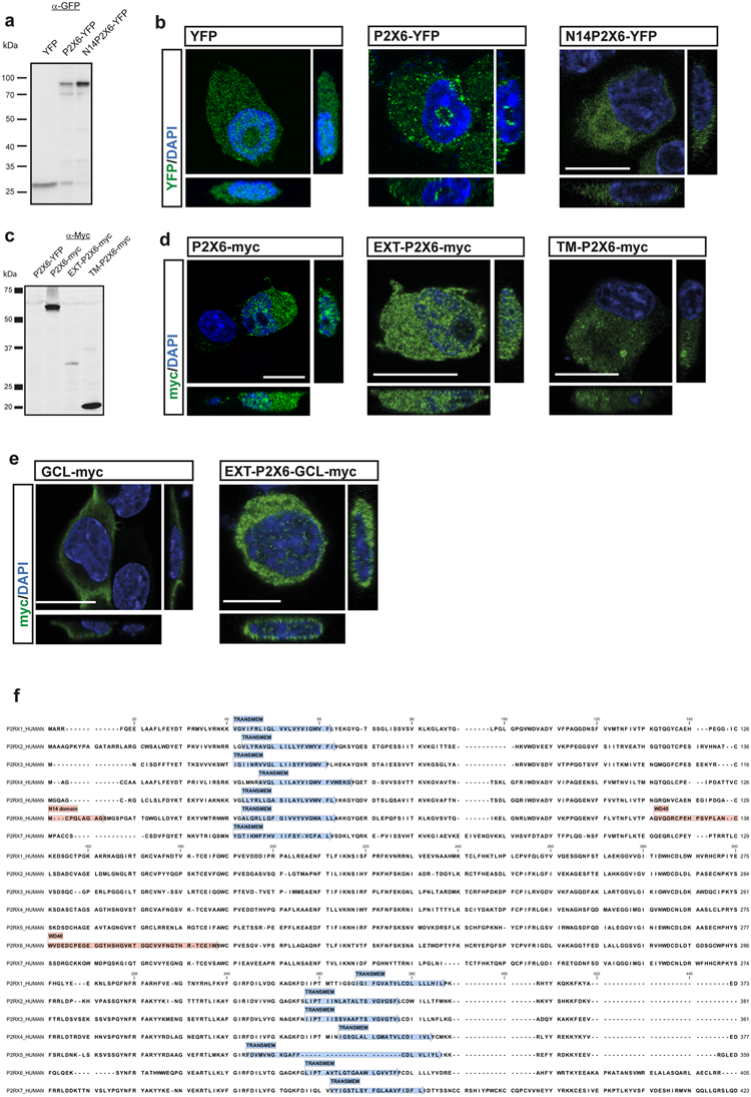
Extracellular region of P2X6 is responsible for nuclear location. (**a**) Western blot of protein extracts from neuroblastoma cells transfected with constructions containing YFP as control, P2X6 and N-terminal defective P2X6 fused with YFP (N14P2X6-YPF). (**b**) Confocal images and orthogonal views of N2a neuroblastoma cells transfected with YFP, P2X6-YFP and N14P2X6-YPF. Subcellular distribution of both YFP and N14P2X6-YFP were exclusively cytosolic, whereas P2X6-YFP shows cytoplasmic and nuclear location. Scale Bar 10 μm. (**c**) Western blots of protein extracts from neuroblastoma cells transfected with constructions containing different P2X6 subregions fused with c-myc epitope and labelled with anti-myc antibody. (**d**) Confocal images and orthogonal views of N2a neuroblastoma cells transfected with P2X6-myc, P2X6 extracellular region (EXT-P2X6-myc), and second transmembrane domain (TM-P2X6-myc). Subcellular distribution of TM-P2X6-myc were exclusively cytosolic, whereas EXT-P2X6-myc shows cytoplasmic and nuclear location. Scale bar 10 μm. (**e**) Neuroblastoma cells transfected with GCL-myc and P2X6 extracellular domain fused with GCL-myc (EXT-P2X6-GCL-myc) constructions show different locations. Subcellular distribution GCL-myc were exclusively cytosolic, whereas EXT-P2X6-GCL-myc shows cytoplasmic and nuclear location. Scale bar 10 μM. (**f**) Protein alignment of human P2X subunits using ClustalX algorithm and sequence analysis by PFAM database of protein families revealed the presence of WD40 domain exclusively in P2X6 subunit.

To identify how P2X6 subunit reaches the nucleus, a new set of molecular tools were developed and assayed in cellular model lacking native P2X6 subunit, the Neuro 2a cell line [26]. First, considering that N-terminal fragment of P2X6 provokes its retention inside the ER [11], we transfected neural cells, with a P2X6-YFP form lacking the N-terminal end (lacking its 10 first hydrophobic residues, MCPQLAGAGSN, N14P2X6-YFP) (Fig 4a). The absence of P2X6 in the nucleus of cells expressing N14P2X6-YFP confirmed that the retention inside the ER is a step required for the following nuclear localization of P2X6 (Fig 4b). Second, P2X6 subunit fragments were cloned by PCR in order to identify the region involved in the guidance of P2X6 subunit into the nucleus (Fig 4c). Our results showed that, whereas the extracellular domain fused with c-myc flag (region between aminoacid 61 to 333, called EXT-P2X6-myc) was capable of reaching a nuclear localization (Fig 4d), the construction that encodes the transmembrane TM2 and the C terminal region fused with c-myc flag (from the aminoacid 334 to 441 called TM-P2X6-myc) presented a cytoplasmic but non-nuclear location. To support these data we created a chimeric protein formed by the fusion of a cytosolic protein, the glutamate-cysteine ligase modifier subunit (GCLm), and EXT-P2X6. This construction changed GCLm distribution from its native cytosolic presence to a nuclear one (Fig 4e).

Analyzing the aminoacid sequence of the extracellular region of P2X6 subunit, we determined that this subunit does not contain a canonical nuclear localization signal (NLS). However, P2X6 is the only P2X subunit that presents an interacting protein motif WD40 in its extracellular domain (Fig 4f).

### Several nuclear proteins co-immunoprecipitate with P2X6 subunit

**Fig 5:**
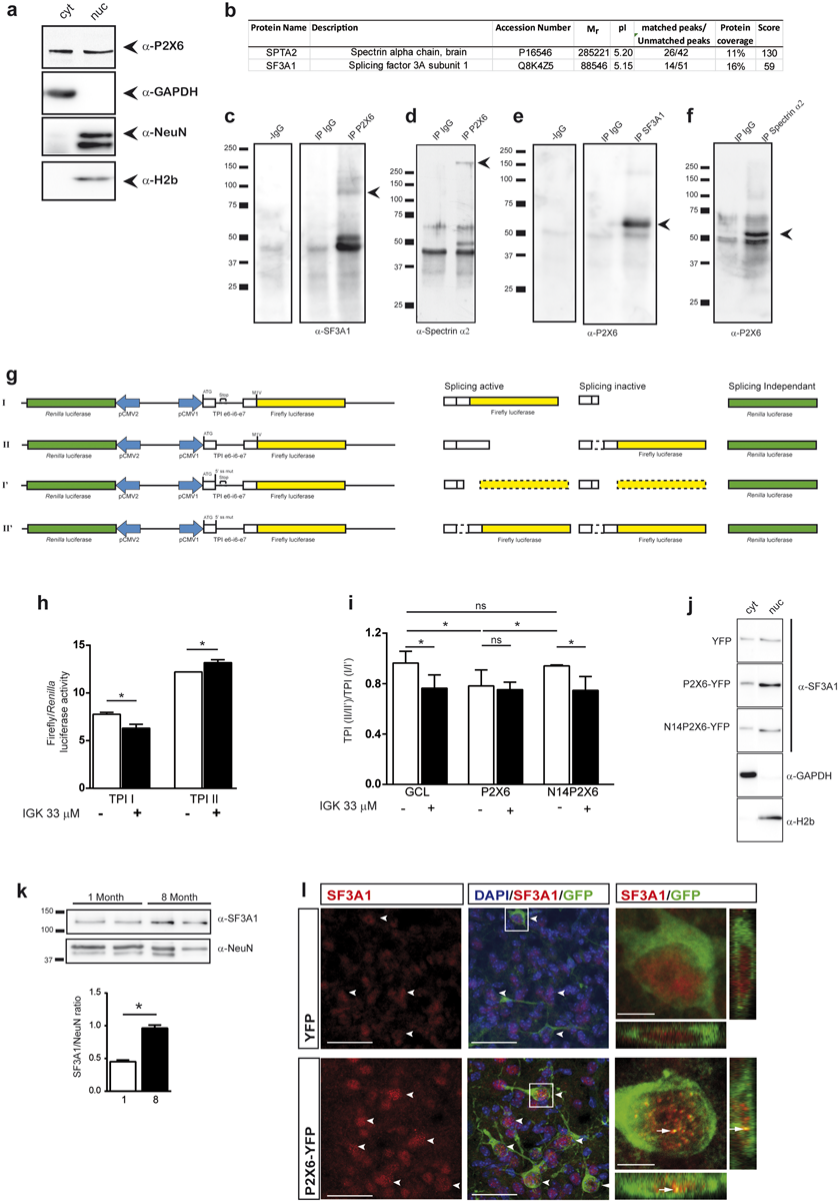
P2X6 subunit interacts with SF3A1 and Spectrin α2 in nuclear extracts from hippocampus of 8 month-old mice and alters splicing efficiency *in vitro*. (**a**) Western blot of subcellular fractions from 8 month-old mice hippocampus. P2X6 subunit was located in both the cytosolic (cyt) and the nuclear fraction (nuc), as identified by localization with either glyceraldehyde-3-phosphate dehydrogenase (GAPDH) or NeuN (neuronal nuclei marker)/histone 2b (H2b), respectively. (**b**) Nuclear extracts immunoprecipitated with anti-P2X6 antibody were resolved in a 2D electrophoresis gel and selected spots were isolated, processed and identified by MALDI/TOF (n = 3 mice). (**c-f**) Nuclear extracts immunoprecipitated with either IgG or anti-P2X6 antibodies were analysed by immunoblotting with antibodies against SF3A1 (**c**) and spectrin α-2(**d**). Nuclear extracts were also immunoprecipitated with spectrin α-2 (**e**) or SF3A1 (**f**) antibodies and immunoblotted with anti-P2X6 antibody. (**g**) Scheme shows four DNA constructions prepared for splicing activity quantitation and their respective protein translations. (**h**) Splicing inhibitor isoginkgetin (IGK) was used to validate the molecular tools employed to quantify the splicing efficiency. As expected TPI I firefly luciferase-dependent luminescence decreases and TPI II firefly luciferase-dependent luminescence increases when splicing is impaired (mean±s.e.m., n = 5 independent experiments, * P<0.05 unpaired Student’s t-test). (**i**) Overexpression of full-length P2X6 subunit reduces splicing activity in neuroblastoma cells compared to either control (GCL) or N14P2X6 transfected cells. The inhibition is similar to those produced by IGK (mean±s.e.m normalized values, n = 3 independent experiments, * P<0.05 unpaired Student’s t-test, ns P>0,05 unpaired Student’s t-test). (**j**) Western blot of subcellular fractions from neuroblastoma cells transfected with YFP, P2X6-YFP or N14P2X6-YFP, showing an increase of SF3A1 in the nuclear fraction only in P2X6-YFP transfected cells. (**k**) Western blot of hippocampal nuclear fractions from 8 month-old mice showing an increase of SF3A1 in the nucleus (n = 4 mice, *P<0.05 unpaired Student’s t-test). (**l**) Developing mice (E14.5) were *in utero* electroporated with YFP or P2X6-YFP and harvested at P0. Brain sections were labeled with antibodies against GFP and SF3A1, showing an increase of SF3A1 in the nucleus of transfected neurons. Arrows heads indicate transfected cells with high SF3A1 expression and full arrows show evident dotted pattern colocalization of SF3A1 and YFP. Scale bar 25 μm. Enlarged view scale 5 μm.

To validate this hypothesis and identify the proteins that are interacting with P2X6 subunit inside the nucleus, we performed co-immunoprecipitation assays with protein extracts of isolated hippocampal nuclei from adult mice. After corroborating the presence of P2X6 subunit in the nuclear fraction (Fig 5a), these extracts were immunoprecipitated with anti-P2X6R antibody or IgG. The eluted product was resolved in a 2D electrophoresis gel. Several spots were isolated, processed and identified by MALDI/TOF (Fig 5b). The nuclear proteins positively identified were spectrin α2 and SF3A1. Corroboration of the specific interaction between both proteins and P2X6 subunit was obtained by reciprocal immunoprecipitation assays performed with specific antibodies against spectrin α2, SF3A1 and p2X6. (Fig 5c–5f). As expected, the nuclear fraction from N2a cells overexpressing full length P2X6-myc, EXT-P2X6-myc or EXT-P2X6-GCL-myc were immunoprecipitated with anti-myc antibody and revealed with anti-SF3a1 antibody, showed a clear enrichment on SF3a1 levels compared with levels detected in same samples non immunoprecipitated with c-myc antibody (S3c Fig). However, this enrichment of SF3a1 levels was missing in nuclear fraction from cells transfected with TM-P2X6-myc (S3c Fig). All immunoprecipitations described were repeated 2 times from at least three independent preparations from hippocampal nuclei or N2a cells.

### The interaction between nuclear P2X6 subunit and SF3A1 modifies splicing efficiency

To investigate the influence of the interaction between P2X6 and SF3A1, we developed a new molecular tool based in triose phosphate isomerase exon 6–7 and intron 6 [21] in-frame with luciferase in two different dual luciferase/renilla reporter vectors, TPI-I and TPI-II, which allow us to evaluate spliceosome activity (Fig 5g). RT-PCR analysis of total RNA from each cell line confirmed that the transcripts from constructs TPI-I and TPI-II were spliced under standard conditions, however when these cell lines were treated with isoginkgetin 33 μM, the transcripts encoded by these constructs were not spliced (S3a Fig). The reliability of this tool was demonstrated by the pharmacological inhibition of the splicing activity with the biflavonoid isoginkgetin, which induced an increase in luminescence signal reported by vector II and a decrease in the signal produced by vector I (Fig 5h). Interestingly, overexpression of P2X6 subunit in neural cells produced both a significant reduction of splicing activity (Fig 5i) and a significant increase in the nuclear levels of SF3A1 (Fig 5j). Nevertheless, significant changes either in splicing activity or intranuclear levels of SF3A1 were not detected in cells transfected with N-14P2X6 or GCL (Fig 5I and 5j).

It is worth noting that, in a similar way as described for P2X6 (Fig 1g), a significant increase in nuclear levels of SF3A1 was also detected in 8 months-old mice with respect to those observed in 1 month-old mice. (Fig 5k). In addition, when we analyzed the expression of SF3A1 in electroporated neurons from in utero electroporation assays, a change in the nuclear distribution pattern of SF3A1 was observed. So, while in non-electroporated or YFP-electroporated cortical neurons, the SF3A showed a homogenous distribution pattern inside the nucleus (Fig 5l), this changed to a dotted pattern in neurons overexpressing P2X6-YFP (Fig 5l). The same change in the nuclear distribution pattern of SF3A1 was observed in N2a cells after they overexpressed P2X6-YPF (S3d Fig). All these data together with the high degree of overlapping between the fluorescence signals from P2X6-YFP and SF3A1, strongly suggests that SF3A1 is being recruited by P2X6 subunit when this reaches the nuclear compartment (Fig 5l and S3d Fig).

## Discussion

In the present work, using immunological techniques as well as the development of molecular biology tools, we were able to demonstrate that P2X6 subunit reaches the nuclear compartment. The nuclear location of P2X6 is favored by the retention that this subunit suffers in the RE through its N-terminal domain (its first N-terminal 10 residues, which are not well conserved in the others P2X subunits). In addition, the change to a nuclear location that the cytosolic protein glutamate-cysteine ligase modifier subunit (GCLm) suffers when it was fused to the extracellular domain of P2X6, constitutes a very solid proof indicating this domain contains a sequence located between the residues 61 and 333 that really bears the capacity to guide a protein toward the nucleus. Moreover, when we performed a more detailed analysis of the extracellular domain of P2X6 it was found that this is the only P2X subunit that possesses a WD40 motif on it. The main function of proteins containing WD40 domains is to coordinate the assembly of multiprotein complex, which serves as scaffolding for protein-protein interactions [27]. The canonical structure of WD40 domain usually contains 4 to 8 WD40 repeats. However, in some cases, proteins with only 6 repeats need to incorporate an additional WD40 motif from a donor protein, as has been reported for Seh1, a component of the mayor subcomplex of the nuclear pore complex (NPC) [28]. So, it is possible to suggest that P2X6 subunit interacts with a nuclear protein that requires a WD40 donor in order to reach cell nucleus. However, although we cannot rule out this hypothesis, we couldn´t find any nuclear pore complex protein interacting with P2X6 subunit. Nevertheless, other nuclear proteins that directly bind to P2X6 subunit were identified, including spectrin α2 and the splicing factor 3A1. But then, how can the P2X6 subunit reach the nucleus? The answer could be found in a peripheral channel which is close to NPC, where transmembrane proteins can move bidirectionally by lateral diffusion [29]. Based on this possibility, we postulate that P2X6 subunits retained in ER could reach the peripheral channel of NPC by lateral diffusion and then translocate to the nucleus. Once inside, P2X6 subunit interacts with spectrin α2 at the inner nuclear envelope, facilitating in this way its retention inside the nucleus. Supporting this hypothesis, we detected an accumulation of P2X6 subunits both in the nuclear envelope of P2X6-YFP transfected hippocampal neurons, as well as associated with the inner nuclear membrane of hippocampal neurons from CA3 region. In addition, considering that both proteins may remain interacting inside de nucleus, the protein complex resulting might act as scaffold able to interact with a wide spectrum of proteins such as RNA processing proteins or structural proteins [30]. Hence, it´s reasonable to think that the interaction between both proteins can favors the nuclear accumulation of P2X6 subunits.

Once inside the nucleus, P2X6 subunit also interacts and recruits the splicing factor 3A1, a component of the SF3A heterotrimeric complex, that is part of the small nuclear ribonucleoprotein particle U2 (U2 snRNP) [13]. Due to the key role that this complex plays on the recognition of the intron branch point (BS) and in the prespliceosome assembly [15], both essential steps to start the splicing reaction [12], it is logical to think that the interaction between SF3A1 and P2X6 subunit inside the nucleus could induce a deep modification in the splicing function. According to this hypothesis, a significant decrease in the splicing activity was observed only in those cells where the nuclear translocation of the P2X6 subunit took place. These results are in concordance with those reported by Tanackovic and Kramer in HeLa cells, showing that the knockdown of whatever subunit that forms the SF3A complex resulted in a general block in splicing activity [31]. But also these results are indicating that the extracellular domain of P2X6 subunit, which contains the WD40 domain, is essential for the interaction between both proteins. Supporting this hypothesis it was observed that two proteins that do no interact in their native form, as the cytosolic GCL protein and SF3a1, are able to interact after the cytosolic protein was fused with extracellular domain of P2X6. Alongside the splicing activity reduction an increase in SF3A1 nuclear levels was detected both on cells overexpressing P2X6 subunit and in adult mice. Interestingly, previous works have reported that the increased expression of SF3A in the cerebellum of mice has been related with the aging associated alterations [16]. In this way it has also been described that in some animal species a deregulation of splicing activity with age takes place [18]. In agreement with this hypothesis, we not only observed that the association between nuclear P2X6 subunit and nuclear envelope, where it is present the spectrin α2, increases with the age, but the number of neurons with nuclear P2X6 accumulation also increases with age. It is noteworthy that alternative splicing of this subunit only takes place in early mouse brain development [32]. The close relationship that appears to exist between the nuclear accumulation of P2X6 subunits and age, and also considering that alteration on splicing activity has been related with a syndrome associated with a premature aging, the Progeria syndrome [33], led us to ask whether the alteration on splicing activity induced by nuclear translocation of P2X6 could be related with senescence. However our results discarded this hypothesis since senescent cells are not enriched in nuclear P2X6 subunit.

Since the nuclear speckles are intra-nuclear structures that have been widely associated with the splicing factors inside the nucleus, initially we thought that P2X6 might be involved in the building of the scaffold of these structures. However, this hypothesis was discarded since positive P2X6 nuclear bodies do not co-localized with nuclear speckles identified by the specific marker sc-35 [34].

Despite the fact that P2X subunits don´t have any known consensus sequence of nuclear localization signal (NLS) on their cloned sequences, the presence inside the nucleus of other P2X subunits, such as the P2X7 subunit, has been already proposed [35]. However, until now, neither the mechanism involved in this phenomenon nor the possible nuclear functions altered by its presence has been described. In the present work we report for the first time that the extracellular domain of P2X6 is able to target this subunit in the nucleus, where P2X6 can interact at least with spectrin α2 and SF3A1. Moreover, the interaction with the SF3A1 subunit negatively regulates the splicing activity, which may be related to P2X6 subunit accumulation during maturation/aging. It is well known that developmental progress is a process related to maturation of cellular function. Thus, the negative modulation that nuclear accumulation of the P2X6 subunit elicits on splicing activity, could explain, at least in part, the events associated with cellular reprogramming of neuronal consolidation.

## Supporting Information

S1 ARRIVE Checklist ARRIVE GUIDANCE. The ARRIVE guidelines are designed to improve the reporting of animal research. This document demonstrates how the ARRIVE guidelines has been used in practice to report animal research, by providing specific information for each point of the checklist.
(PDF)

S1 Fig Age related accumulation of P2X6 receptor in CA3. **(a)** Quantification of nuclear P2X6 positive cells per slice in hippocampus of young vs adult mice (mean±s.e.m., n = 3 mice, ** P<0.01 unpaired Student’s t-test). (**b**) Immunohistochemical analysis of brain section from 8 and 18 months-old mice. Immunohistochemistry against P2X6 subunits (brown) in hippocampal slices. Scale bar 200 μm. Insets depict enlarged views (40X magnification) of delimited area.
(TIF)

S2 Fig Single channel microphotographs for subcellular distribution of native P2X6 subunits in hippocampal neurons in vitro. (**a,b**) Immunofluorescence images of 3(**a**) and 18(**b**) days-old primary cultures of hippocampal neurons labeled with antibodies against P2X6 and β-III Tubulin. Nuclei were counterstained with DAPI. Confocal image and orthogonal views of hippocampal neurons show that P2X6 immunostaining is localized in ER compatible location and inside the nucleus, and shows a more dotted pattern as days in culture passed. Scale bar 5 μm. (**c-e**) Immunofluorescence images 16–18 days-old primary cultures of hippocampal neurons labeled with antibodies against P2X6 and NeuN (**c**), fibrillarin (**d**) or SC35 (**e**). Nuclei were counterstained with DAPI. Confocal image and orthogonal views of hippocampal neurons show that P2X6 immunostaining is localized in ER compatible location and inside the nucleus without colocalization with NeuN (**c**), fibrillarin (**d**) or SC35 (**e**). Scale bar 5 μm.
(TIF)

S3 Fig Analysis of constructions for the measure of splicing activity and SF3A1 immunoprecipitation with different P2X6 constructions. **(a),** RT-PCR analysis of total RNA isolated from the indicated transfections and/or isoginkgetin (33 μM) treated cells and plasmid TPI I (size control for unspliced transcripts) as described in S1 Materials and Methods. (**b),** Splicing activity measured with constructions TPI I and TPI II in N2a cells co-transfected with P2X6 chimeric constructs and their respective controls, as well as N2a cell transfected only with TPI constructions (* p<0.05 YFP vs P2X6-YFP, # p<0.05 GCL-myc vs P2X6-myc, One way ANOVA with Tukey’s multicomparison post-test). **(c),** Nuclear extracts from N2a cells transfected with P2X6-myc, P2X6 extracellular region (EXT-P2X6-myc), second transmembrane domain (TM-P2X6-myc) and GCL-P2X6-EXT-myc were immunoprecipitated with either IgG or anti-myc antibodies and were analyzed by immunoblotting with antibodies against SF3A1. (**d**) Transfected N2a cells labeled with GFP and antibodies P2X6 and SF3A1, showing a change in the expression pattern of SF3A1 in the nucleus of cells. Scale 10 μm.
(TIF)

S1 Materials and Methods RT-PCR protocol. (DOCX)
