# Supplementary figures and images for: Age-Related Nuclear Translocation of P2X6 Subunit Modifies Splicing Activity Interacting with Splicing Factor 3A1
Source: PLoS One. 2015 Apr 13;10(4):e0123121. doi: 10.1371/journal.pone.0123121 (PMC4395284; doi:10.1371/journal.pone.0123121)

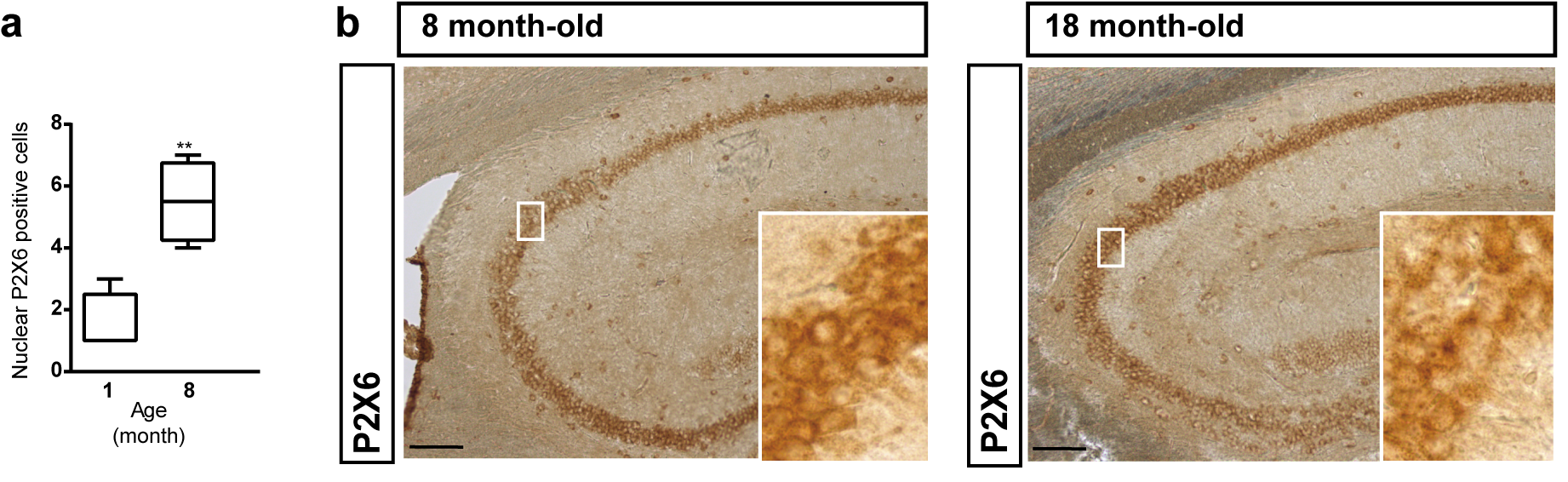

Supplement: S1 Fig — (a) Quantification of nuclear P2X6 positive cells per slice in hippocampus of young vs adult mice (mean±s.e.m., n = 3 mice, ** P<0.01 unpaired Student’s t-test). (b) Immunohistochemical analysis of brain section from 8 and 18 months-old mice. Immunohistochemistry against P2X6 subunits (brown) in hippocampal slices. Scale bar 200 μm. Insets depict enlarged views (40X magnification) of delimited area. (TIF) [file pone.0123121.s002.tif]

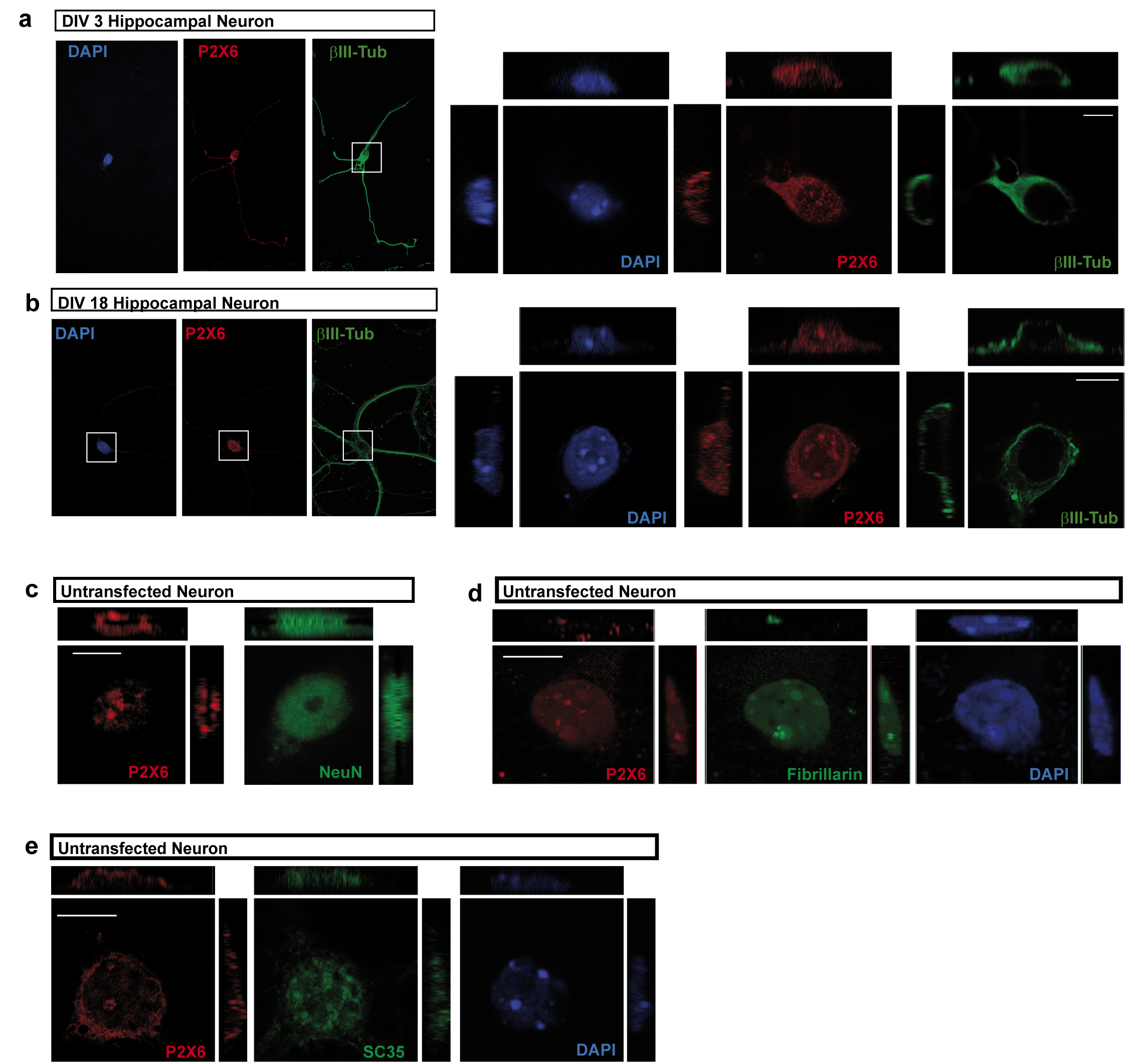

Supplement: S2 Fig — (a,b) Immunofluorescence images of 3(a) and 18(b) days-old primary cultures of hippocampal neurons labeled with antibodies against P2X6 and β-III Tubulin. Nuclei were counterstained with DAPI. Confocal image and orthogonal views of hippocampal neurons show that P2X6 immunostaining is localized in ER compatible location and inside the nucleus, and shows a more dotted pattern as days in culture passed. Scale bar 5 μm. (c-e) Immunofluorescence images 16–18 days-old primary cultures of hippocampal neurons labeled with antibodies against P2X6 and NeuN (c), fibrillarin (d) or SC35 (e). Nuclei were counterstained with DAPI. Confocal image and orthogonal views of hippocampal neurons show that P2X6 immunostaining is localized in ER compatible location and inside the nucleus without colocalization with NeuN (c), fibrillarin (d) or SC35 (e). Scale bar 5 μm. (TIF) [file pone.0123121.s003.tif]

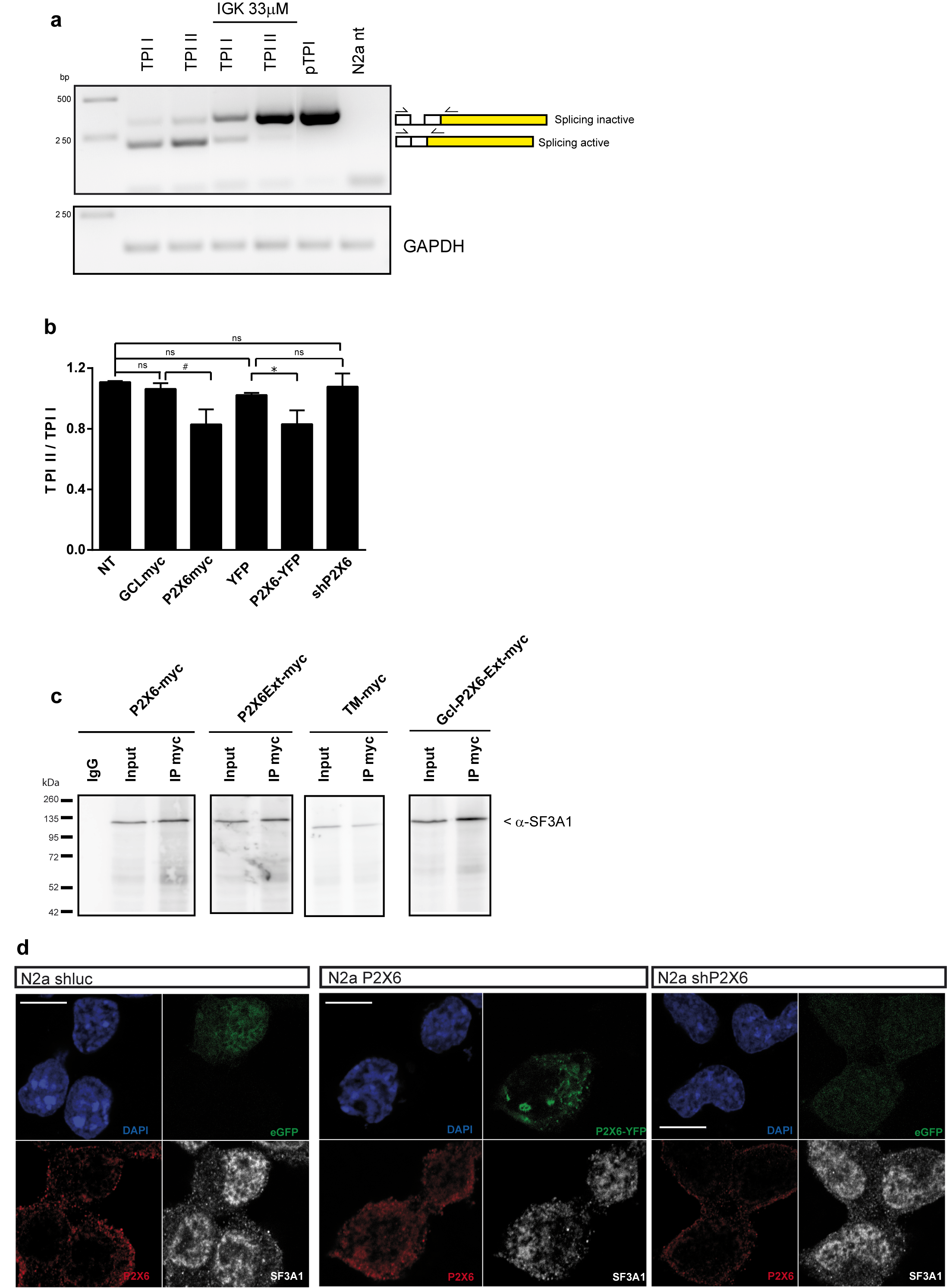

Supplement: S3 Fig — (a), RT-PCR analysis of total RNA isolated from the indicated transfections and/or isoginkgetin (33 μM) treated cells and plasmid TPI I (size control for unspliced transcripts) as described in S1 Materials and Methods. (b), Splicing activity measured with constructions TPI I and TPI II in N2a cells co-transfected with P2X6 chimeric constructs and their respective controls, as well as N2a cell transfected only with TPI constructions (* p<0.05 YFP vs P2X6-YFP, # p<0.05 GCL-myc vs P2X6-myc, One way ANOVA with Tukey’s multicomparison post-test). (c), Nuclear extracts from N2a cells transfected with P2X6-myc, P2X6 extracellular region (EXT-P2X6-myc), second transmembrane domain (TM-P2X6-myc) and GCL-P2X6-EXT-myc were immunoprecipitated with either IgG or anti-myc antibodies and were analyzed by immunoblotting with antibodies against SF3A1. (d) Transfected N2a cells labeled with GFP and antibodies P2X6 and SF3A1, showing a change in the expression pattern of SF3A1 in the nucleus of cells. Scale 10 μm. (TIF) [file pone.0123121.s004.tif]
