# Supplementary material for: Age-Related Nuclear Translocation of P2X6 Subunit Modifies Splicing Activity Interacting with Splicing Factor 3A1
Source: PLoS One. 2015 Apr 13;10(4):e0123121. doi: 10.1371/journal.pone.0123121 (PMC4395284; doi:10.1371/journal.pone.0123121)
Supplement: S1 Materials and Methods — (DOCX) [file pone.0123121.s005.docx]

### Materials and methods S1. RT-PCR

Total RNA was purified from cultured N2a cells using a Speedtools total RNA Extraction Kit (Biotools) according to the manufacturer´s instructions. After digestion with TURBO DNase (Ambion) 1 µg of total RNA was quantified and reversed transcribed using M-MLV reverse transcriptase, 6 µg of random primers and 350 µM dNTPs (all from Invitrogen). PCR were carried out using DNA AmpliTools Master Mix (Biotools) in 25 µl final volume with oligonucleotides designed for Exon 6 of TPI (5’- TAA ACT TAA GCT TCA GCG CCT CGG-3’) and luciferase cDNA (5’- GGC CCT TCT TAA TGT TTT TGG-3’).
